# Room temperature terahertz semiconductor frequency comb

**DOI:** 10.1038/s41467-019-10395-7

**Published:** 2019-06-03

**Authors:** Quanyong Lu, Feihu Wang, Donghai Wu, Steven Slivken, Manijeh Razeghi

**Affiliations:** Center for Quantum Devices, Department of Electrical Engineering and Computer Science, Northwestern University, Evanston, IL 60208 USA

**Keywords:** Mid-infrared photonics, Quantum cascade lasers, Semiconductor lasers

## Abstract

A terahertz (THz) frequency comb capable of high-resolution measurement will significantly advance THz technology application in spectroscopy, metrology and sensing. The recently developed cryogenic-cooled THz quantum cascade laser (QCL) comb has exhibited great potentials with high power and broadband spectrum. Here, we report a room temperature THz harmonic frequency comb in 2.2 to 3.3 THz based on difference-frequency generation from a mid-IR QCL. The THz comb is intracavity generated via down-converting a mid-IR comb with an integrated mid-IR single mode based on distributed-feedback grating without using external optical elements. The grating Bragg wavelength is largely detuned from the gain peak to suppress the grating dispersion and support the comb operation in the high gain spectral range. Multiheterodyne spectroscopy with multiple equally spaced lines by beating it with a reference Fabry-Pérot comb confirms the THz comb operation. This type of THz comb will find applications to room temperature chip-based THz spectroscopy.

## Abstract (web summary)

Terahertz frequency combs are highly desired for applications in precision measurements, sensing, spectroscopy and metrology. Here the authors demonstrate the room-temperature chip-based THz frequency comb using nonlinear frequency generation from a mid-infrared quantum cascade laser comb.

## Introduction

Optical frequency comb (OFC) is a laser source with its spectrum consisting of a series of discrete, evenly-spaced frequency lines.^1^ Driven by their applications in spectroscopy, metrology, etc., many techniques have been demonstrated to generate OFCs, including mode-locked lasers^2–5^, and Kerr-nonlinearity based micro-resonators^6^. For mid-infrared (mid-IR) to terahertz (THz) range (λ ~ 3–300 μm) that cannot be easily accessed with the above techniques, difference-frequency generation^7^, optical parametric oscillation^8^, and photoconductive antenna^9^ have been developed. However, these comb sources involve elaborate optical setups.

Quantum cascade laser (QCL) based on InP substrate has become the leading mid-IR laser source thanks to the dramatic advances in material growth control, power efficiency, and widely tailorable frequency emission^10–12^. Owing to its unique intersubband transition and fast phonon scattering, the mode-locking is very challenging for QCLs^13^. On the other hand, under engineered dispersion and gain designs, the QCL multimode operation can be locked into comb modes via four-wave mixing (FWM) enabled by a third-order *χ*^(3)^ nonlinearity^14^. After significant advancements in power efficiency^15,16^ and dispersion management^17–20^, mid-IR QCL frequency combs have emerged as appealing comb sources with reproducible phase relations^21,22^. Based on similar nonlinear effects and dispersion engineering, GaAs-based THz QCLs are also capable of frequency comb operation^23–27^. Despite their potential applications of spectroscopy^28^ and imaging^29^, THz-QCL comb development is not yet comparable to its mid-IR counterpart due to, in part, their cryogenic operation.

Here, we report a room temperature THz frequency comb based on difference-frequency generation from a mid-IR QCL. A largely frequency-detuned distributed-feedback (DFB) grating is integrated into the QCL cavity to provide a single mode at *λ*_1_ and a harmonic-state comb at *λ*_2_. The THz comb is intracavity generated via down-converting the multimode comb with the DFB mode into the THz range. The device emits in a harmonic-state frequency comb with a mode spacing of 157 GHz in a frequency range of 2.2–3.3 THz and continuous wave power up to 5 μW at room temperature and exhibits improved frequency stability which is desired for chip-based THz spectroscopy.

## Results

### Largely-detuned grating design

The QCL structure is based on a single-stack active region structure designed for a wavelength at *λ* ~ 7.8 μm and was grown on a semi-insulating (SI) InP substrate. The active region was engineered with high second-order nonlinear susceptibilities for THz difference-frequency generation (DFG)^30^. To down-convert a mid-IR comb to the THz range, a single mode or another mid-IR comb at a different frequency must be generated simultaneously, as has been done externally using two separate laser sources^7^. Since the dual wavelength operation with a THz frequency spacing is not easy to achieve in QCLs due to the gain competition^31^, many wavelength selection mechanisms have been used, including the composite DFB^32^ and the dual-section DFB^33^ for single mode THz emissions, the dual-section DBR-DFB (DBR: distributed-Bragg reflector)^34^ and the multi-section DBR-SGDFB (SG: sampled grating)^35^ for tunable THz emissions, and the weakly-coupled DFB for the broadband THz emission^36^. However, none of these techniques can emit a comb and single mode at the same time. This is because these mode selecting structures not only normally suppress the comb operation with the strong distributed feedback mechanism for single mode operation but can also introduce a significant amount of dispersion into the laser cavity.

**Fig. 1:**
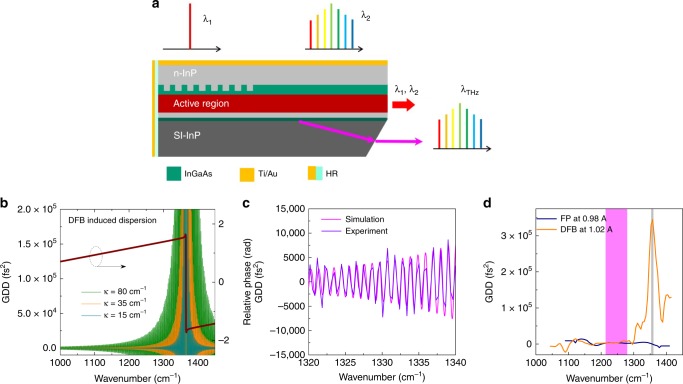
Largely-detuned DFB grating design for THz comb nonlinear generation from a mid-IR QCL. **a** Schematic of largely detuned DFB QCL design for THz frequency comb operation. **b** Calculated GDD spectra at different coupling coefficient *κ* and the DFB induced relative phase spectrum for *κ* = 15 cm^−1^. **c** measured high-resolution GDD spectra of a DFB with *κ* = 35 cm^−1^ and *L* = 1.5 mm and its comparison with the calculated results. **d** Measured low-resolution GDD spectra of the DFB and FP devices. The gray and pink bars indicate grating and comb spectral positions

The dispersion of a typical DFB grating that is used for single mode operation is systematically investigated theoretically and experimentally. Figure 1b plots the relative phase and group delay dispersion (GDD) of a DFB grating in the QCL-based device as shown in Fig. 1a. A transfer matrix method is used in the simulation. The grating exhibits an abrupt change in relative phase Δ*φ* with Δ*φ* ≈π and a shape dispersion peak at the grating Bragg wavelength. The DFB-induced dispersion is highly oscillatory with the repetition frequency determined by the length of grating section. This is confirmed experimentally by the measured high-resolution dispersion transformed from the interferogram of the subthreshold spontaneous spectrum (Supplementary Fig. 1), as shown in Fig. 1c. Clearly, this oscillatory feature could completely change the laser cavity dispersion if it was designed at or near the gain peak as in most of the previous DFB designs used for THz generation^32,33^. Nevertheless, the DFB-induced dispersion decreases as the detuning from the Bragg wavelength increases and as the lowering of the grating coupling coefficient *κ*.

In this work, a largely detuned DFB grating is proposed to realize single mode and comb operation in the same cavity. The DFB wavelength position is detuned by ~80–90 cm^−1^ with respect to the comb emission wavelength to minimize the effect of the DFB structure on laser dispersion. Meanwhile, the grating coupling strength (*κL*, *L* = grating length) is set to be 3–5 to secure the single mode operation for the largely detuned DFB design. In the comb operating spectral range of 1240–1280 cm^−1^, the calculated DFB dispersion is limited to ±1500 fs^2^. A low-resolution dispersion analysis was performed for the DFB and a reference Fabry-Pérot (FP) laser with an identical geometry to retrieve the total dispersions of the devices. Similar dispersions of ~3000 fs^2^ in the comb operating range were observed for both devices. This reveals that limited dispersions are induced for the designed DFB device near the gain peak, which indicates that the grating design will not pose a negative effect on the four-wave mixing for comb operation.

### On-chip generation of room temperature THz harmonic-state frequency comb

**Fig. 2:**
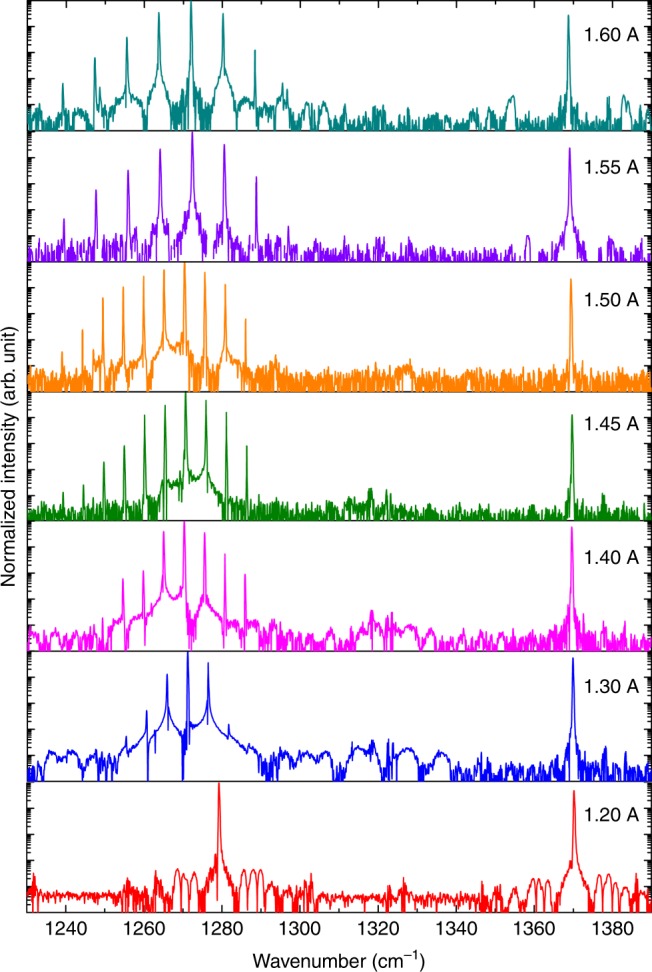
On-chip generation of single mode and multimode comb from a single mid-IR QCL. Lasing mid-IR spectra of the 4-mm long DFB QCL evolving with currents from 1.2 to 1.60 A at room temperature in continuous wave operation

The DFB and FP devices with the identical geometry are tested using a Bruker Fourier transform infrared (FTIR) spectrometer. The FP device goes through different lasing states, from single mode, to harmonic state, hybrid harmonic-density state, and eventually density state comb operation with a mode spacing defined by one free spectral range (FSR) of the cavity (Supplementary Fig. 2). Narrow beatnote spectra were observed for the FP device with dense-state emissions in a current range *I* = 1.42–1.55 A with linewidths <1 kHz. On the contrary, the DFB device emits a single mode at *λ*_1_ = 7.25 μm defined by the grating period and a pronounced multimode emission at *λ*_2_ = 7.81 μm simultaneously, as shown in Fig. 2. Instead of emitting dense-state fundamental comb as the FP device, the detuned DFB device produces stable multimode emissions with a mode spacing corresponding to 14 times the free spectral range (FSR) of the laser cavity for currents *I* ≤ 1.5 A and 22 times the FSR at higher currents. This shows the device skips many longitudinal modes and favors a few, powerful modes separated by many FSRs.

Note that the DFB grating is not acting as a mode selection element for this multimode emission as in semiconductor mode locked lasers^37^. This is because the sideband of the DFB reflection has a different mode spacing of ~1 cm^−1^ and their reflection near the gain is about three-order of magnitude weaker than that at Bragg wavelength. Since the QCL is a semiconductor laser with an ultrafast gain recovery time (~0.5 ps), the incoherent gain induced by the SHB effect allows multiple modes to reach threshold, and parametric gain induced by the population pulsation effect suppresses the nearby modes and enhances the largely spaced modes^38^. The impact of the DFB element is twofold. The single mode emission resulted from the DFB section forms a spatial population grating in the cavity and induces an incoherent gain for multimode operation near the gain peak at higher currents. On the other hand, the beating between the single DFB mode and the multimode introduces additional population pulsation nonlinearity which in turn contributes to the mode skipping of the multimode emission in the working current range.

This harmonic comb phenomenon has also been recently observed from QCLs with different coating conditions at different wavelengths^38,39^, which indicates the underlying mechanism is an intrinsic property of QCLs, i.e., the fast gain recovery time associated with the intersubband transition. However, the operation range of harmonic comb is limited in a narrow current range for the HR-coated QCLs as shown in Fig. 2S in this work and ref. ^39^ and uncoated QCLs in ref. ^38^. The harmonic comb can be enhanced by increasing the asymmetry of the cavity with HR–AR coating to weaken the population grating effect and force the device undergo only the AM instability^38^ or by pumping the device with an external laser^40^. The detuned DFB design in this work provides another approach to effectively improve the current dynamic range of the comb operation by enhancing the SHB effect and population pulsation effect.

**Fig. 3:**
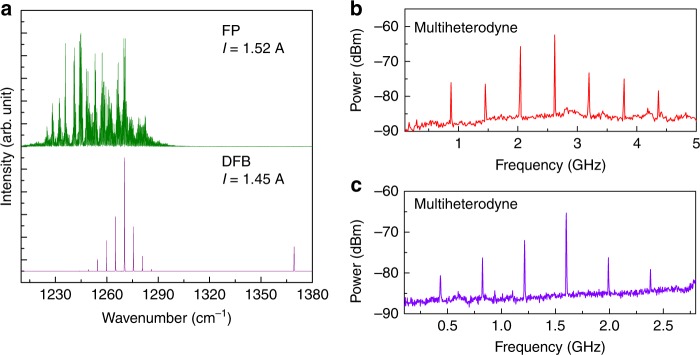
Multi-heterodyne characterizations based on the DFB and FP QCL combs. **a** Lasing spectrum of the FP comb at 1.52 A and the DFB comb at 1.45 A recorded with the FTIR, respectively. Multi-heterodyne beating of the FP comb at 1.52 A and the DFB comb at 1.45 A (**b**), and the DFB comb at 1.55 A (**c**). Total acquisition time: 100 ms

Since the mode spacing of the harmonic state comb with many FSRs exceeds the bandwidth of conventional mid-IR detectors, a multi-heterodyne beating experiment between the harmonic state DFB device and a reference comb device can be performed to assess the comb operation for the DFB device^41^. Here, the FP device is biased into a fundamental comb operation regime to serve as the reference comb. The two devices were focused on a high-bandwidth quantum well infrared detector (QWIP) (Bandwidth >20 GHz) for heterodyne measurement and the heterodyne signal was recorded with a spectrum analyzer. In parallel, the beams from the two devices are sent to the FTIR for spectral characterization. The electrical and temperature tuning rate for the carrier offset frequency of the FP device can be induced directly from the repetition frequency and lasing frequency tuning rates, which is estimated to be 103.8 MHz/mA and −0.98 GHz/K, respectively. (Supplementary Note 3). Since the repetition frequency *f*_rep,2_ for the harmonic comb is so large that it cannot be measured directly with the spectrum analyzer. In the experiment, the bias on the DFB device is fixed or roughly tuned, while the FP current is finely tuned to shift the heterodyne frequencies into the spectral range of 0–6 GHz that is of interest. The FP device was tuned to a current of 1.52 A to lase in the fundamental comb state, while the DFB device was biased to 1.45 A to lase in the multimode harmonic state, as shown in Fig. 3a. The heterodyne spectra were recorded with a spanning range of 5 GHz, a resolution bandwidth (RBW) of 300 kHz, and scan time of 100 ms. Figure 3b shows that equally-spaced beating modes are observed on the multi-heterodyne spectrum with a mode spacing of 580 MHz, corresponding to the beating of the central 7 modes from the DFB device with the FP comb modes. The linewidth for each beat signal is ~300 kHz which is limited by the RBW. The current of the DFB device is then increased steadily from 1.55 A to lase in the harmonic state with a different mode spacing of 22 FSR. The recorded heterodyne spectrum with a mode spacing of 390 MHz is shown in Fig. 3c. Note that the heterodyne mode spacing is merely determined by the frequency difference between the mode spacing of the harmonic comb and the integer multiple of FP mode spacing (See Method section “Multi-heterodyne measurement”). Given the comb operation of the FP device at the biased current, this multi-heterodyne experiment verified the harmonic comb operation of the DFB device.

**Fig. 4:**
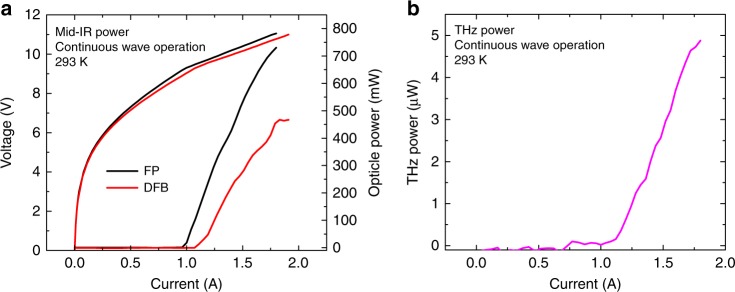
Power characterizations of mid-IR and THz comb emissions. **a** Power-current-voltage (PIV) characterizations of the FP and DFB QCL combs with 4-mm long cavity and 9-μm ridge width at room temperature continuous wave operation. **b** THz power characterization of the DFB QCL comb at room temperature continuous wave operation

Optical power-current-voltage (*P*-*I*-*V*) characterizations for both devices under continuous wave operation at 293 K are performed. The DFB device emits a continuous wave power upto 0.46 W with a threshold current density of 2.7 kA/cm^2^, while the FP device emits upto 0.73 W with a threshold current density of 2.5 kA/cm^2^, as shown in Fig. 4a. The slope efficiency is 1.18 W/A, compared with the 0.81 W/A for the DFB device. The reduced slope efficiency of the DFB device compared with the FP device is expected since the FP device, featuring dense-state comb operations at higher currents, is able to extract more gain and power than that of the DFB device. This can also be understood phenomenally from the light-intensity distribution point of view, wherein the nodal regions of the intensity standing wave induced by strong SHB effects have reduced gains. The power is reduced for about 40% for the DFB compared with FP device, which is attributed to the strong SHB effect. A theoretical modal addressing the SHB-induced power reduction is developed in the Supplementary Note 5.

**Fig. 5:**
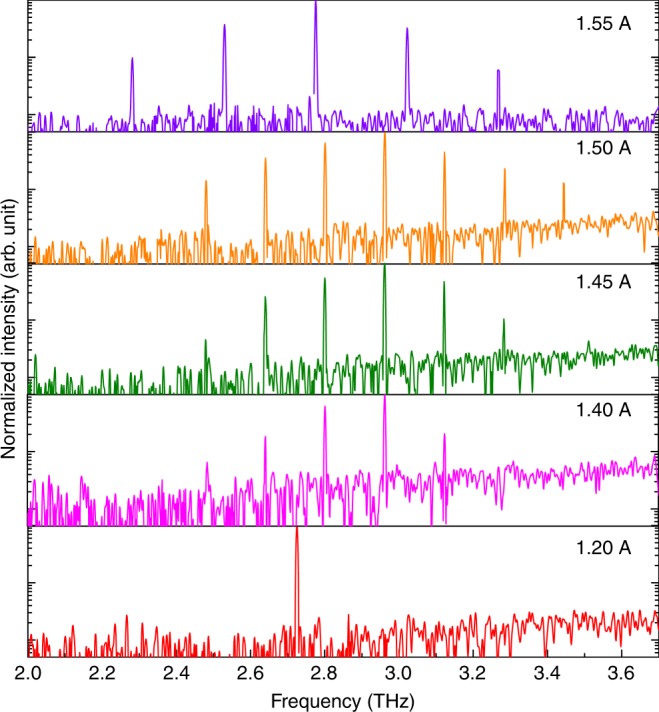
On-chip generation of THz frequency comb from a mid-IR QCL. Lasing THz spectra of the DFB QCL comb evolving at different currents from 1.20 to 1.55 A at room temperature continuous wave operation

The dual wavelength operation of the DFB device enables the second-order difference-frequency generation that can transfer the mid-IR comb into the THz range with *λ*_THz_ = 1/(1/*λ*_1_ − 1/*λ*_2_). The SI InP substrate allows extraction of THz radiation through the substrate via the Čerenkov phase matching scheme for broadband THz generation^30,33^. The THz emitting spectra were measured with the FTIR spectrometer equipped with a silicon bolometer. Multimode harmonic comb operation in the THz range was observed in a wide current range, as shown in Fig. 5. The mode spacing is 157 GHz for currents between 1.3–1.5 A and 245 GHz for currents >1.5 A. These spacings correspond to 14 FSR and 22 FSR mode spacings determined by the mid-IR pumping combs. The tuning rates of the emitting frequency and carrier frequency for the THz comb are investigated and estimated to be −0.56 cm^−1^/A and 6.9 MHz/mA. Both parameters are over ten times smaller than the mid-IR comb counterparts. (Supplementary Note 3). This is because the two mid-IR pumping sources are simultaneously generated from the same chip, and share similar frequency tuning rates, which improves the stability of the THz comb. The coherence of the comb phase ought to mainly observe the phase relation of the mid-IR comb with additional minor modulation from the nonlinear susceptibility. (Supplementary Note 4). A similar dual-comb heterodyne setup equipped with a fast THz detector can be used to further verify the coherence of the THz comb. Thanks to the mid-IR harmonic state comb operation with a limited mode number, the converted THz light intensity is concentrated on a limited number of comb modes with a side mode suppression ratio upto 20 dB. The THz power was measured with a calibrated Golay cell detector. The DC injecting current is modulated with a low-frequency modulation to match the testing condition of the detector. The DFB device emits a continuous THz power upto 5 μW, as shown in Fig. 4 b, which indicates that average power of 1 μW for each THz mode is achieved.

The demonstrated largely detuned DFB design enables new features that are extremely useful for mid-IR and THz comb generation. First, it enhances harmonic comb operation with an increased current dynamic range and its reproducibility by introducing strong SHB and population pulsation effects. Pronounced harmonic frequency comb operation has been repeatedly observed from multiple DFB devices. This approach could be further improved to explore monolithic control and tuning of the harmonic comb emission. Here, two DFB sections with different wavelength and coupling can be designed in the waveguide and independently pumped. While the largely detuned DFB section secures the single mode operation, a new DFB section can be designed with a wavelength near the gain peak so that a much smaller coupling coefficient κ can be used to reduce its effect on the dispersion. As a result, the single mode emission from this new DFB section could be used as an optical seeding to monolithically control and tune the mid-IR and THz harmonic comb emission. More importantly, the dual wavelength operation of DFB and harmonic comb operation enables chip-based THz comb operation at room temperature without any external optical elements. The next step of our research will be focused on the realization of fundamental THz frequency comb operation by DFG in QCL. This can be achieved by reducing the SHB effect either by reducing the DFB coupling strength or using an anti-reflection coating on the front facet. This will also enable more comb modes to extract gain for higher power operation. Octave-spanning THz combs could be also potentially achieved by using a broadband QCL design^42^ or simply pushing the DFB wavelength closer to the gain peak to reducing the starting frequency of the comb. This type of device design strategy will further shed light on frequency comb generation in the shortwave range (<4 μm) via sum frequency generation^43^ where the QCL cannot readily emit a frequency comb directly owing to its large dispersion in this spectral range. Finally, the demonstrated harmonic frequency comb in mid-IR and THz ranges with large frequency spacing and powerful comb modes will significantly reduce the complexity of the arrayed waveguide grating design for wavelength demultiplexing of the comb in the application of communication and optical arbitrary waveform generation^44^.

## Discussion

In conclusion, we report a room temperature THz harmonic frequency comb at 3.0 THz based on difference-frequency generation from a mid-IR QCL comb. A largely detuned distributed-feedback grating is integrated into the QCL cavity to provide single mode operation, as well as an enhanced spatial hole-burning effect for multimode comb operation. The THz comb signal is intracavity generated via down-converting the mid-IR multimode comb with a DFB mode into the THz range of 2.2–3.3 THz. Multi-heterodyne spectroscopy with multiple equally spaced lines by beating it with a reference FP QCL comb verifies the THz comb operation. This device design not only elucidates interesting design schemes for the control of the harmonic comb in mid-IR range, but also enables a chip-based THz comb with improved stability at room temperature which is appealing to the applications like spectroscopy.

## Methods

### Growth and fabrication

The QCL structure presented in this work is based on a strain-balanced Al_0.63_In_0.37_As/Ga_0.35_In_0.65_As material system with Ga_0.47_In_0.53_As inserts^30^ grown by gas-source molecular beam epitaxy (MBE) on a SI InP substrate. The growth was started with a 200-nm InGaAs layer (Si, ~1 × 10^18^ cm^−3^) and a 3-μm InP buffer layer (Si, ~4 × 10^16^ cm^−3^). The laser core consisted of a 40-stage strain-balanced single-phonon resonance (SPR) structure. The average doping of the active region is ~2.0 × 10^16^ cm^−3^. The MBE growth ended with a 400-nm-thick InGaAs grating layer (Si, ~2 × 10^16^ cm^−3^) and a 10-nm-thick InP cladding layer (Si, ~2 × 10^16^ cm^−3^). Part of the sample is patterned with a DFB grating designed with a period of 1.15 μm and depth of 250 nm via e-beam lithography and dry etching. After the grating patterning, metal organic chemical vapor phase deposition (MOCVD) was then used for the growth of a 3.5-μm-thick InP cladding layer (Si, ~2–5 × 10^16^ cm^−3^) and 1-μm-thick InP cap layer (Si, ~1 × 10^19^ cm^−3^). The doping level of the cladding cap layers is adjusted to minimize the material dispersion.

The wafer was processed into buried ridge waveguides with a ridge width of 8–9 μm. Four millimeter long DFB and FP devices were directly coated with a Y_2_O_3_/Au HR coating on the back facet using an ion-beam deposition (IBD) system and e-beam evaporation system. The front facet is uncoated. The length of DFB section is 1.5 mm. The SI-InP substrate was then polished into 30° to outcouple the THz light satisfying the Čerenkov phase-matching condition. Testing was done on a thermoelectric cooler (TEC) stage at room temperature (293 K). For continuous wave measurement, the optical power was measured with a calibrated thermopile detector placed directly in front of the laser facet for mid-IR power measurement. A Golay cell detector (Microtech Instruments) was used for THz power measurement. In order to be compatible with Golay cell detection, the DC current was modulated with a low-frequency pulse to modulate the THz signal. The power value was not corrected for collection efficiency.

### Multi-heterodyne measurement

The two combs are first collimated by 1-inch f/0.5 anti-reflection (AR) coated aspheric lenses. Both combs are separated by a 50–50 beam splitters and focused onto a fast (>20 GHz, Debut Optoelectronic) liquid-nitrogen cooled QWIP operating at 8.2 μm. A neutral density filter with transmission <1% is used to attenuate the power focused on to the detector and to avoid any possible saturation of the detector. The heterodyne signal is then acquired using a spectrum analyzer (Agilent-E4407B). Both combs are driven with low noise current drivers (Wavelength electronics QCL 2000) with a specified average current noise density of 4 nA/$\sqrt {{\mathrm{Hz}}}$. The temperature fluctuations of the lasers were also reduced to less than 10 mK by using a low thermal drift temperature controller (wavelength electronics PTC10K-CH) with a 50 k Ω thermistor.

For the FP and DFB devices, each comb mode can be expressed as:

1 $$f_m = f_{{\mathrm{ceo}},1} + m\,f_{{\mathrm{rep}},1}$$

2 $$f_n = f_{{\mathrm{ceo}},2} + n\,{\mathrm{ }}f_{{\mathrm{rep}},2}$$

where *f*_rep,1_, and *f*_rep,2_ are the repetition frequencies and corresponds to the spacing between the modes, and *m* and *n* are integer numbers. The offset frequencies *f*_ceo,1_ and *f*_ceo,2_ correspond to a common offset shared between all modes. The repetition frequency *f*_rep,1_ equals one FSR for the FP device in fundamental comb mode, and *f*_rep,2_ equals multiple FSR for the DFB device in harmonic comb operation. The heterodyne beating spectra is generated when *f*_rep,2_ is slightly different from the integer multiple of *f*_rep,1_ by a certain frequency Δ*f*_0_.

3 $$f_{{\mathrm{rep}},2} = {\mathrm{\Delta }}f_0 + l\,f_{{\mathrm{rep}},1}$$

where *l* is an integer number. Thus, each mode for the heterodyne spectrum is:

4 $$f_{\mathrm{h}} = f_{\it{n}}-f_{\it{m}} = f_{{\mathrm{ceo}},{\mathrm{h}}} + nf_{{\mathrm{rep}},{\mathrm{h}}}$$

with

5 $$f_{{\mathrm{ceo}},{\mathrm{h}}} = f_{{\mathrm{ceo}},2} - f_{{\mathrm{ceo}},1}$$

and

6 $$f_{{\mathrm{rep}},{\mathrm{h}}} = (l - m/n)f_{{\mathrm{rep}},1} + {\mathrm{\Delta }}f_0$$

are the offset frequency and the repetition frequency of the heterodyne spectrum.

## Supplementary information

Supplementary material

## Data Availability

The data that support the findings of this study are available from the corresponding author upon reasonable request.
