# Supplementary material for: Room temperature terahertz semiconductor frequency comb
Source: Nat Commun. 2019 Jun 3;10:2403. doi: 10.1038/s41467-019-10395-7 (PMC6546691; doi:10.1038/s41467-019-10395-7)
Supplement: Supplementary file 1 — Supplementary material [file 41467_2019_10395_MOESM1_ESM.pdf]

Supplementary information for

Room temperature terahertz semiconductor frequency comb

Lu, *el al.*

Supplementary Note 1 | GDD measurements of the FP and DFB devices

The GDD spectra for FP and DFB are acquired by using a Fourier transform technique[1,2]. The characterization is done by measuring the spontaneous emission of the device at subthreshold CW operation with a FTIR. The subthreshold emission spectra show the DFB grating induces a strong peak with a detuned frequency of Δ= 80 cm^{-1} with respect to the gain peak (Supplementary Figure 1(a)). A spectral gap of 3.5 cm^{-1} on the side of the DFB wavelength is also observed, which corresponds to a coupling coefficient of κ~35 cm^{-1}. The corresponding interferogram of the subthreshold spectrum contains the relative phase information. The secondary burst from left in Supplementary Figure 1(b) corresponds to the moving mirror position at which the interferometer optical path difference matches a single round trip optical distance within the laser cavity. The Fourier transform of this burst will provide the relative phase information of the cavity mode. The GDD spectra presented in Supplementary Figure 1(c) are obtained by performing the second derivative to the relative phase spectra. The main point here is that the burst is truncated with two different interferogram lengths, as indicated in Supplementary Figure 1(b). The longer interferogram corresponds to a spectral resolution of 0.3 cm^{-1} that contains the information about the oscillatory dispersion induced by DFB, as shown in Fig. 1(c), but the spectrum also contains increased noise as shown in Supplementary Figure 1(c) (blue curve). The shorter interferogram corresponds to a spectral resolution of 4 cm^{-1} and contains much less noise and only reveals the information about the dispersion induced by the gain, material, and waveguide as shown in Supplementary Figure 1(c) (orange curve). Clearly, the designed DFB waveguide exhibits limits GDD near the gain peak induced by the grating section.

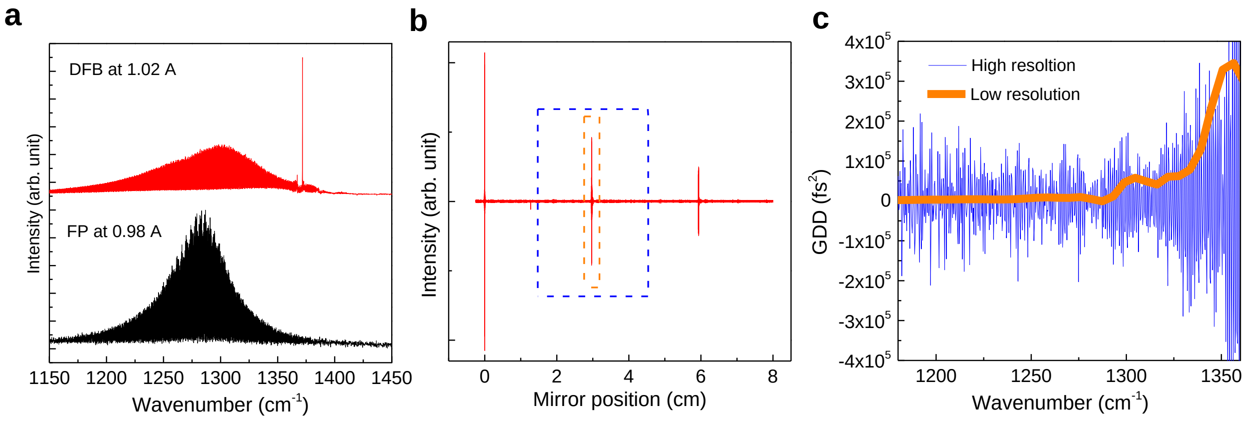

Supplementary Figure 1 | GDD measurement of the DFB and FP devices. (a) Measured subthreshold emission spectra for the DFB and FP devices. (b) Interferogram for the DFB spectra. The blue and orange boxes indicate the truncated mirror positions for high- and low-resolution Fourier transforms. (c) Generated high and low resolution GDD spectra. The high-resolution spectrum contains the high-frequency information with a much higher noise level than the low-resolution spectrum.

Supplementary Note 2 | Spectral and beatnote evolutions of the FP device

The purpose of this section is to provide further information on the spectral and beatnote evolutions of the FP QCL reference device from single mode to dense state, as shown in Supplementary Figure 2. Clearly, the laser went through different laser states: single mode (0.98 - 1.0 A), harmonic state (1.2 A), hybrid harmonic-density state (1.3-1.4 A) and density state (above 1.50 A). The harmonic state features a mode spacing of 5 FSR at 1.2 A. The beatnote characterization was performed by focusing the laser beam into a QWIP, and the beatnote spectra was recorded with a spectrum analyser. Weak and unstable beatnote is observed for the hybrid harmonic-density state in 1.3-1.4 A current range, which indicates that stable fundamental comb operation is not yet reached. Narrow and stable electrical beatnote state was observed in the density state with current 1.42-1.55 A with beatnote full-width at half maximum (FWHM) <1 kHz. The beatnote gets broader as the current increases above 1.55 A, which indicates high phase noise at higher currents.

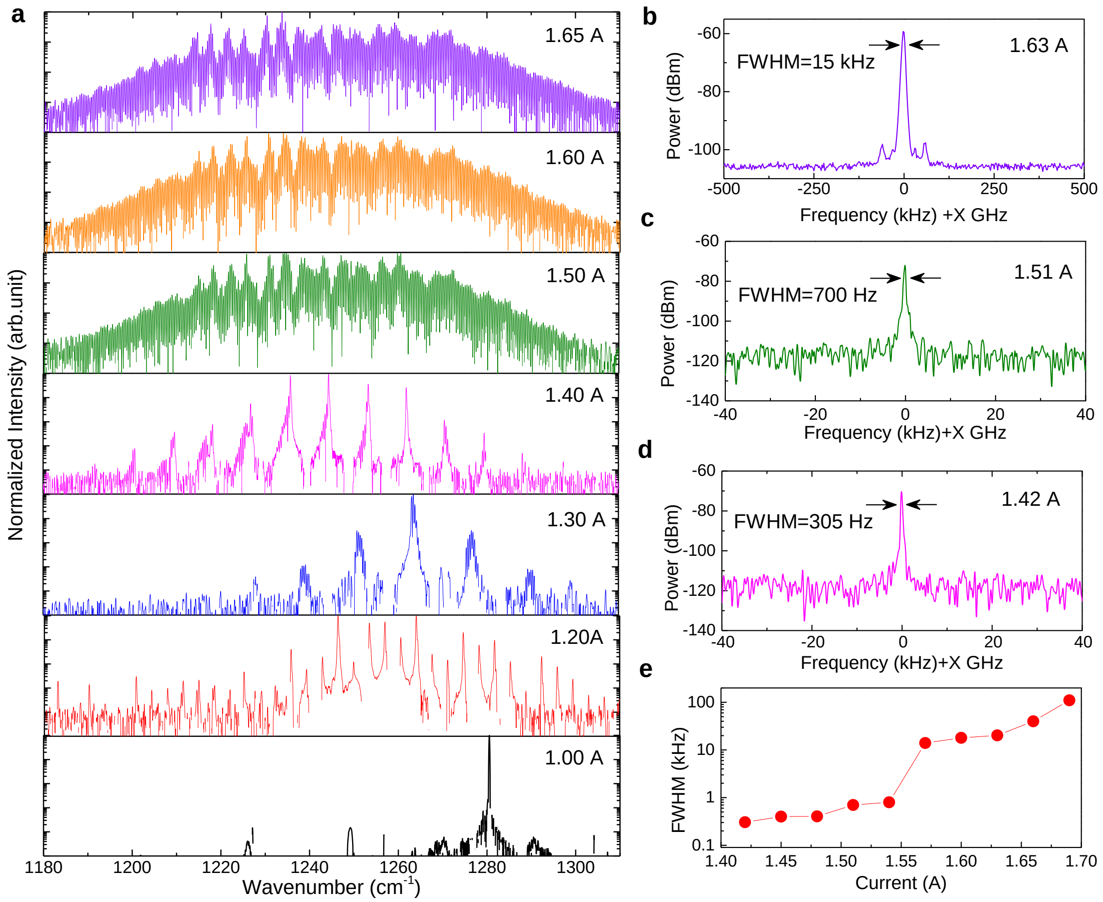

Supplementary Figure 2 | Spectral and beatnote evolutions of the FP device. (a) Lasing mid-IR spectra of the FP QCL with a cavity of 4 mm at different currents. The beatnote spectra at different currents (b) and its linewidths as a function of currents at 293 K (c). X=11.220, 11.227, 11.235 GHz denotes the beatnote frequency position. The frequency spanning and RBW are 1 MHz and 1 kHz for (b), and 400 kHz and 300Hz for (c) and (d).

Supplementary Note 3 | Frequency tuning characterizations of the FP and DFB devices

The carrier offset frequency $f_{ceo,1}$ of the FP QCL comb cannot be precisely determined because the laser is in free-running condition. However, its tuning rate as a function of current or temperature can be determined by comparing the change of the repetition frequency $f_{rep,1}$ and the lasing frequency f_m. Supplementary Figure 3(a) shows the lasing spectra of the FP device at different currents. Given the measured electrical tuning rate of -75 kHz/mA, and -4.92 cm^{-1}/A for $f_{rep,1}$ and f_m, the electrical tuning rate for $f_{ceo,1}$ is estimated to be 107 MHz/mA. This tuning rate of the carrier frequency is also close to that reported in Ref. 3. Clearly, the shift of the offset frequency $f_{ceo,1}$ is much larger than that of the repetition frequency, this is mainly because the offset frequency tuning is expected to be N times higher than the repetition frequency tuning, where N is the number of comb lines. Similarly, consider the measured temperature tuning rate of -1.5 MHz/K, and -0.2 cm^{-1}/K for $f_{rep,1}$ and f_m, the temperature tuning rate for $f_{ceo,1}$ is estimated to be -0.91 GHz/K.

Since the repetition frequency $f_{rep,2}$ for the harmonic comb is so large and cannot be measured directed with the spectrum analyser, we will use Eqs. (1-6) in the Method section to estimate the tuning rate for $f_{rep,2}$ and $f_{ceo,2.}$ In the experiment, the bias on the DFB device is fixed while the tuning FP current to tune the heterodyne signal. The corresponding tuning rate for the heterodyne frequency is measured to be 20 MHz/mA (Supplementary Figure 3(b)). From Eqs. 4-6, a tuning rate of -355 kHz/mA is obtained for $f_{rep,h}$. As a result, we induced the repetition tuning rate of -1.39 MHz/mA for harmonic comb $f_{rep,2}$, which is determined by: $f_{rep,2} = f_{rep,h} + (m/n)\, f_{rep,1}$. Considering the electrical tuning rate of -5.6 cm^{-1}/A for the mid-IR harmonic comb, its carrier offset frequency $f_{ceo,2}$ tuning rate is estimated to be 173 MHz/mA from Eq. (2).

For the intracavity generated THz comb, using the measured electrical tuning rate of -6.16 cm^{-1}/A for the DFB wavelength, with the information of the tuning rates of $f_{rep,2}$ and $f_{ceo,2}$ for the harmonic comb, the tuning rates of the emitting frequency and carrier frequency for the THz comb are estimated to be -0.56 cm^{-1}/A and 6.9 MHz/mA. Both parameters are over ten times smaller than the mid-IR comb counterparts. This is attributed to the fact that the two mid-IR pumping sources from the same chip sharing similar frequency tuning rates, which improves the stability of the THz comb.

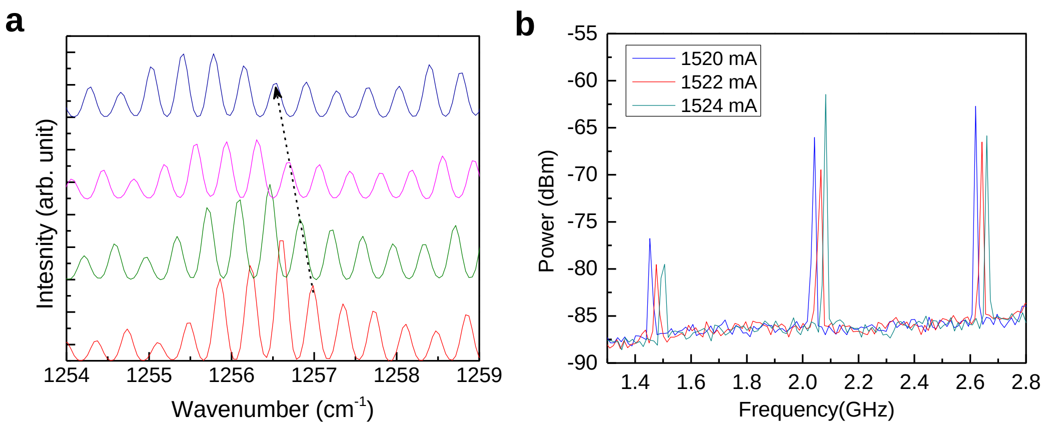

Supplementary Figure 3 | Frequency tuning characterizations of FP and heterodyne spectra. (a) Lasing mid-IR spectra of the FP QCL with increase currents from 1500 mA to 1590 mA with a step of 30 mA. The arrow denotes the spectral tuning with the increasing currents. (b). Heterodyne beating spectra at different injecting currents on the FP device while DFB current is fixed.

Supplementary Note 4 | Coherence study of THz combs based on DFG QCLs

The coherence between the harmonic state modes and the DFB mode is another important aspect in the verification of the THz comb emission by difference frequency generation. In the experiment, we use the Čerenkov phase matching scheme to achieve phase matching between the harmonic comb modes and the DFB mode. The Čerenkov condition is fulfilled as long as the THz refractive index of the substrate n_{THz} is larger than the group index n_g of the mid-IR pump modes. Then the Čerenkov emission angle is given by the following $\theta_{\check{C}}=\cos^{-1}(n_g/n_{\mathrm{THz}})$. The electromagnetic waves of the harmonic comb and the DFB can be written as:

$$\mathbf{E}_c(\vec{r},t) = \sum_n E_n e^{i\mathbf{k}_c\mathbf{r}-i2\pi f_n t} = \sum_n |E_n| e^{i\mathbf{k}_c\mathbf{r}-i2\pi f_n t+i\phi_n} \quad (1)$$

$$\vec{E}_0(\vec{r},t) = |E_0| e^{i\mathbf{k}_0\mathbf{r}-i2\pi f_0 t+i\phi_0} \quad (2)$$

Here, ϕ_n is the phase of comb mode n, and ϕ_0 is the phase of the DFB mode which is a fixed value in a definite condition. $\mathbf{k}_c$ and $\mathbf{k}_0$ are the comb and DFB wave vectors. In the process of difference frequency generation, the nonlinear THz polarization wave at $f_{\mathrm{THz}}=f_0-f_n$ in a QCL waveguide can be written as[4]:

$$\mathbf{P}^{(2)}(\vec{r},t) = \varepsilon_0 \sum_n \chi^{(2)}_{n,0} |E_n||E_0| e^{i(\mathbf{k}_0-\mathbf{k}_c)\vec{r}-i2\pi(f_0-f_n)t+i(\phi_0-\phi_n)} \quad (3)$$

$$=\varepsilon_0 \sum_n \left|\chi^{(2)}_{n,0}\right| |E_n||E_0| e^{i\mathbf{k}_{\mathrm{THz}}\vec{r}-i2\pi f_{\mathrm{THz}}t-i\left(\phi_n-\phi_0-\phi_\chi\right)} \quad (4)$$

Here, the THz wave vector fulfils the Čerenkov phase matching condition $|\mathbf{k}_{\mathrm{THz}}| = |\mathbf{k}_0 - \mathbf{k}_c|/\cos(\theta_c)$. ϕ_χ is the phase introduced by second order nonlinear susceptibility $\chi^{(2)}$:

$$\chi^{(2)}_{n,0}(f_{\mathrm{THz}}) = \Delta N \frac{e^3}{\varepsilon_0 \hbar^2} \frac{z_{12} z_{23} z_{31}}{(\omega_{\mathrm{THz}} - \omega_{23} + i\Gamma_{23})} \left[\frac{1}{(\omega_0 - \omega_{13} + i\Gamma_{31})} - \frac{1}{(\omega_n - \omega_{12} + i\Gamma_{21})} \right] \quad (5)$$

This nonlinear susceptibility and its induced phase are slowly varying parameters and can be computed using the related parameters[5].

Clearly, the phase relation of the THz comb mainly inherits that of the mid-IR comb with additional minor modulation from the nonlinear susceptibility. Based on intersubband transition, both QCL comb and QCL DFB have been demonstrated with quantum-limited linewidths[6,7], which indicates the high coherence of the two type of devices. The intracavity generated THz comb based on the largely detuned DFB design in this work should have similar or even better coherence than the mid-IR QCL comb in free running condition, as the results of the improved stabilities for the THz emitting frequency and carrier offset frequency presented in Note 3.

Supplementary Note 5 | Spatial-hole burning effect on the power and efficiency performance

In the laser structure for THz comb generation, single mode operation by the DFB structure forces a spatial modulation on the laser even at multi-modes operation. This can be modeled as a spatial hole burning effect. In this case, the slop of the *PIV* curve of the laser will be considerably decreased. By comparing the slope efficiency with and without DFB structure, the experimental results agree well with the following calculations.

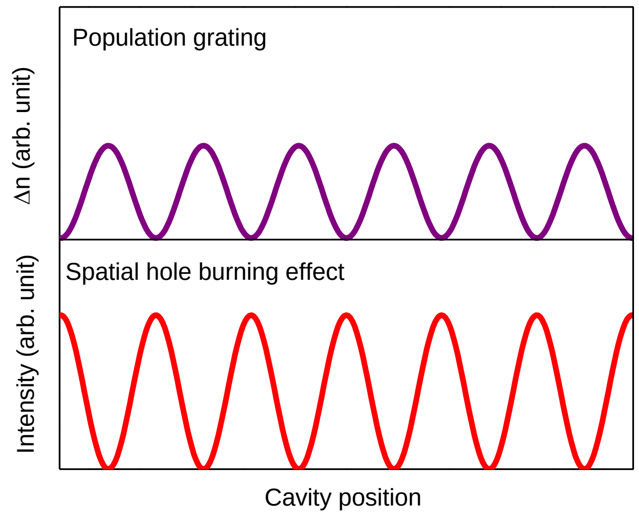

Supplementary Figure 4 | Schematic of spatial hole burning effect and the induced population grating. (a) Schematic of spatial hole burning effect in a laser cavity due to standing wave effect. (b) The population grating, a spatial modulation of the population inversion, formed by the spatial hole burning effect.

Without spatial hole burning effect:

If there are no interference between the two intracavity counterpropagating traveling waves of intensity I_+ and I_-, spatial hole burning effect can be ignored. The total intensity would be $I=I_+ + I_- = 2I_+ = 2I_-$ and the laser steady-state regime could be obtained by considering that this total intensity saturates the gain. Therefore, the output power can be described by the equation below[8]:

$$I_{\text{out}}^{0} = \frac{T}{2} I_{\text{sat}}(r-1) \tag{6}$$

Where r is the ratio between pump currents and threshold currents, T is the transmittance of laser facet, I_{sat} is the saturated intensity.

With spatial hole burning effect:

When two counterpropagating light waves with intensity of I_+ and I_- are superimposed, a standing-wave interference pattern is formed with the period of the half wavelength. This is so called SHB effect, as shown in Supplementary Figure 4 (lower panel). In a QCL gain medium, this has two effects. First, it forms a population grating in the medium and cause the gain saturation to lasing mode in the antinodes of the standing wave, as shown in Supplementary Figure 4 (upper panel). SHB makes the gain for other modes no longer clamped above the threshold and favors multimode operation, as discussed in the main text of the paper. Second, laser amplification is dominated by the excitation density in the antinodes, while the nodes are not excited by the light wave. This intuitively reduces the power and efficiency and many techniques has been developed to mitigate the SHB effect for power efficiency enhancement. A detailed analysis is presented in the following section.

Considering in a laser cavity, the intensities of the two counterpropagating light waves are I_+ and I_-. The total intensity is no longer constant along the cavity ($2I_+$ or $2I_-$). It becomes standing waves: $I = 4|\mathcal{A}|^2 \sin^2 kz$. Where $\mathcal{A}$ is the amplitude of electric field, k is the wave vector and z is the distance along the FP cavity, so the energy density is: $E_{\text{standing}}(z) = 4E\sin^2(\frac{n_0\omega}{c}z)$ where n_0 is the refractive index and c is the speed of light. The saturation of the population inversion then is a function of z[8,9]:

$$\frac{\Delta n(z)}{\Delta n_0} = \frac{1}{1+E_{\text{standing}}(z)/E_{\text{sat}}}$$

(7)

Deriving the laser output power in the presence of spatial hole burning by adapting the following equation from standard rate equation:

$$\frac{dE}{dt} = -\frac{E}{\tau_{\text{cav}}} + \frac{\sigma c}{n_0} \Delta n E(z)$$

(8)

For the case with spatial hole burning, where τ_{cav} is the photon lifetime in the cavity, σ is absorption cross section of the laser cavity. The energy $\mathcal{W}$ stored inside the cavity can be integrated along *z* direction:

$$\frac{d\mathcal{W}}{dt}\Big|_{\text{gain}} = \frac{c}{n_0} \sigma S \int_0^L \frac{\Delta n_0 E_{\text{standing}}(z)}{1 + E_{\text{standing}}(z)/E_{\text{sat}}} dz \quad (9)$$

After integration, we can get the analytical solution:

$$\frac{d\mathcal{W}}{dt}\Big|_{\text{gain}} = \frac{c}{n_0} \sigma S \Delta n_0 E_{\text{sat}} L (1 - \frac{1}{\sqrt{1 + 4E/E_{\text{sat}}}}) \quad (10)$$

Above threshold, the gain of the laser should be clamped to its losses:

$$\frac{d\mathcal{W}}{dt}\Big|_{\text{losses}} = \frac{2EV_{\text{cav}}}{\tau_{\text{cav}}} \quad (11)$$

leading to:

$$\frac{E}{E_{\text{sat}}} = \frac{r}{2} \left(1 - \frac{1}{\sqrt{1 + 4E/E_{\text{sat}}}}\right)$$

(12)

Here, V_{cav} is volume of the gain medium. By only keeping the physically meaningful solution we get:

$$E = \frac{E_{\text{sat}}}{2} (r - \frac{1}{4} - \sqrt{\frac{r}{2} + \frac{1}{16}})$$

(13)

Finally, the laser output intensity can be expressed as following:

$$I_{\text{out}}^{\text{SHB}} = \frac{T I_{\text{sat}}}{2} (r - \frac{1}{4} - \sqrt{\frac{r}{2} + \frac{1}{16}})$$

(14)

So, the slop of the P-I curve gives by:

$$\frac{\partial I_{\text{out}}^{\text{SHB}}}{\partial r} = \left(1 - \frac{1}{4\sqrt{\frac{r}{2}+\frac{1}{16}}}\right) \quad .$$

(15)

Compare with the case without spatial burning effect where the slop is: $\frac{\partial I_{\text{out}}^{0}}{\partial r} = 1 > 1 - \frac{1}{4\sqrt{\frac{r}{2}+\frac{1}{16}}}$, we can get the conclusion that with spatial hole burning effect the slop of *P-I* curve is smaller than the case without spatial hole burning effect. Here we considered the counterpropagating intensity are equal $I_+ = I_-$ which exhibits the strongest hole burning effect case. If the laser end mirrors are not symmetric ($I_+ \neq I_-$), the spatial hole burning is intermediate between these two cases.

Supplementary References

1. Villares, G. *et al.* Dispersion engineering of quantum cascade laser frequency combs. *Optica* **3**, 252-258 (2016).
2. Lu, Q. Y. Wu, D. H., Slivken, S., Razeghi, M. High efficiency quantum cascade laser frequency comb. *Sci. Rep.* **7**, 43806 (2017).
3. Burghoff, D. *et al.* Optomechanical control of quantum cascade laser frequency combs. *Proc. SPIE* **10939**, 109391E-1(2019).
4. Vijayraghavan, K. *et al.* Broadly tunable terahertz generation in mid-infrared quantum cascade lasers. *Nat. Commun.* **4**, 2021 (2013).
5. Lu, Q. Y., Wu, D. H., Sengupta, S., Slivken, S., & Razeghi, M. Room temperature continuous wave, monolithic tunable THz sources based on highly efficient mid-infrared quantum cascade lasers, *Sci. Rep.* **6**, 23595 (2016).
6. Cappelli, F., Villares, G., Riedi, S. & Faist, J. Intrinsic linewidth of quantum cascade laser frequency combs. *Optica* **2**, 836 (2015).
7. Bartalini, S. *et al.* Measuring frequency noise and intrinsic linewidth of a room-temperature DFB quantum cascade laser. *Opt. Express* **19**, 17996–18003 (2011).
8. Svelto, O. Principles of Lasers, *Plenum Press*, New York (1998).
9. Huang, J. & Casperson, L. W. *Optical and Quantum Electronics* **25**, 369-390 (1993).
